# TNF-α inhibitor therapy can improve the immune imbalance of CD4+ T cells and negative regulatory cells but not CD8+ T cells in ankylosing spondylitis

**DOI:** 10.1186/s13075-020-02226-8

**Published:** 2020-06-19

**Authors:** Mingcan Yang, Qing Lv, Qiujing Wei, Yutong Jiang, Jun Qi, Min Xiao, Linkai Fang, Ya Xie, Shuangyan Cao, Zhiming Lin, Yanli Zhang, Liudan Tu, Minjing Zhao, Yunfeng Pan, Ou Jin, Jieruo Gu

**Affiliations:** Department of Rheumatology and Immunology, Third Affiliated Hospital of Sun Yat-Sen University, 600 Tianhe Road, Guangzhou, 510630 Guangdong China

**Keywords:** Ankylosing spondylitis, CD4+ T cells, CD8+ T cells, Negative regulatory cells, TNF-α inhibitor

## Abstract

**Background:**

Studies into ankylosing spondylitis (AS) and its relationship with immune imbalance are controversial, and the correlation between the efficacy of TNF-α inhibitor and changes in immune imbalance is unclear.

**Methods:**

A total of 40 immune cells were tested with flow cytometry, and the results of 105 healthy control (HC) subjects, 177 active-stage AS patients, and 23 AS cases before and after 12 weeks of TNF-α inhibitor therapy (Anbainuo) were analyzed.

**Results:**

Compared with the HC group, the proportion of immune cells, such as naïve and central memory CD4+T cells, in AS increased (*P* < 0.0001), but effector memory and terminally differentiated CD4+T cells were decreased (*P* < 0.01 and 0.0001, respectively). Naïve, central memory, and effector memory CD8+T cells were increased (*P* < 0.0001, 0.001, and 0.01, respectively), but terminally differentiated CD8+T cells were decreased (*P* < 0.0001). Th1 cells (helper T cells-1), Tfh1 cells (follicular helper T cells-1), Tc1 cells (cytotoxic T cells-1), and Tregs (regulatory T cells) were lower (*P* < 0.01, 0.05, 0.0001, and 0.001, respectively), but Th17 cells, Tfh17 cells, and Tc cells were higher (*P* < 0.001, 0.0001, and 0.001, respectively). The proportions of total B cells and class-switched B cells were increased (*P* < 0.05), but non-switched B cells, plasma cells, memory B cells, and immature Bregs (regulatory B cells) were lower (*P* < 0.01, 0.0001, 0.0001, and 0.0001, respectively). After Anbainuo therapy, the percentage of naïve CD4+ T cells had decreased (*P* < 0.05) but Tregs and B10 cells (IL-10-producing regulatory B cells) had increased (*P* < 0.01 and 0.05, respectively), and the increase in Tregs was positively correlated with the decrease in C-reactive protein (CRP) (*r* = 0.489, *P* = 0.018).

**Conclusions:**

We found that active-stage AS patients have an immunity imbalance of frequency involving multiple types of immune cells, including CD4+T cells, CD8+T cells, Th cells, Tfh cells, Tc cells, Tregs, Bregs, and B cells. TNF-α inhibitor Anbainuo can not only help to inhibit disease activity but can also improve the immune imbalance of CD4+ T cells and negative regulatory cells in frequency. But CD8+ T cells have not been rescued.

## Introduction

As the prototype of spondyloarthritis, ankylosing spondylitis (AS) is a chronic inflammatory disease that affects the spine and sacroiliac joints. The pathogenesis of AS can be attributed to both hereditary and environmental factors [1]. The pathogenesis of AS has still remained unclear, but a strong association with the HLA-B27 [2] and a perpetual activation of both the innate and the adaptive immune systems [1], in particular of the IL-23/IL-17 axis and of Th1 effector T cell lineage with the overproduction of tumor necrosis factor-α (TNF-α), are considered to be key steps [1]. Chronic hyperactivation of T lymphocytes and subsequent skewing of functional subgroups within CD4+ T and CD8+ T cells have been supported by some human studies [3, 4]. On the other hand, regulatory T cells (Tregs) display decreased prevalence in the blood of AS patients suggesting that their lack may contribute to the pathogenesis of the disease [5]. However, the data on Tregs have been controversial in AS patients [6]. Studies on acquired immunity to AS have suggested that T cell-mediated immune regulation may also play an important role. Nevertheless, only limited data are available on the phenotypic and functional status of B cells in AS [7, 8]. The ability of B cells to negatively regulate cellular immune responses and inflammation has been explored, and the concept of regulatory B cells (Bregs) has emerged. Indeed, defective suppressive functions of Bregs cell subsets have been observed in several chronic inflammatory diseases [9, 10], but studies into Bregs cell activity in AS patients have been scattered. Some small sample research studies showed regulatory B cells to have a defective function in AS patients but observed no significant change in cell frequency [11].

Given that immune cells in AS have imbalance, manipulating immune cells may be a new therapeutic strategy for treating AS. Therefore, exploring factors that influence immune cell number and function will play a critical role in understanding the pathogenesis of these diseases and identifying new treatment strategies. TNF-α inhibitor has been shown to have an anti-inflammatory effect in treating AS [12], but its potential beneficial effects on the bone structure are still challenging to identify and its mechanisms remain unclear. Although targeting of TNF-α is very effective in AS, around one third of treated patients show only a poor response. The symptoms would recur in the vast majority of patients if the drug is stopped; it is important to know the type and degree of the alterations in the immune system developing during the anti-TNF therapy. On the other hand, with respect to adverse reactions and the high costs of anti-TNF agents leading to high economic burden for the health care systems, it is desirable to stratify patients according to treatment predictors prior to biological therapy. In terms of the immune cell subset distribution, we assume that the major mechanism of action of anti-TNF therapy is to obtain inhibition of the inflammatory process, not the restoration of the activated immune system from active disease to a state similar to healthy individuals. We conducted a prospective study of subset distribution of CD4+/CD8+ T cells and negatively regulate immune cells including Tregs and Bregs by identifying the phenotype, and also analyzed the changes in the proportion of lymphocyte subsets in order to indirectly understand the differentiation status, degree of failure, and cell activity of various cell subsets. That will help us to further clarify changes of the immune system caused by AS and to explore resistance that could contribute to relapse after treatment.

## Materials and methods

### Patients and controls

The Ethics Committee approved this study of the Third Affiliated Hospital of Sun Yat-Sen University, Guangzhou, China. Besides, informed consent was obtained from all patients and all HCs.

All of the AS patients in our study had high disease activity (Ankylosing Spondylitis Disease Activity Score, ASDAS ≥ 1.3), were diagnosed with AS according to the Modified New York Criteria (mNY) [13], and must not have received any biological treatment in the 3 months leading up to the study. Inclusion criteria for control subjects were over the age of 18 years, no current or chronic medication intake, and no known disease or condition. Both AS and HC subjects must not have received a vaccination or suffered an infection in the 3 months leading up to the study.

Peripheral blood lymphocytes were tested with flow cytometry. Parameters of disease activity, including ASDAS and C-reactive protein (CRP), were also registered. No statistically significant differences were observed in terms of age or gender between AS patients and HCs in the two comparison phases. In the primary screening phase of the study, 67 AS patients and 50 HCs were included. In the expanded validation phase, 110 AS patients and 55 HCs were included.

### TNF-α inhibitor (Anbainuo)-treated AS patients

All patients fulfilling the modified New York criteria [13] who were eligible for TNF-α inhibitor treatment due to persistently high disease activity (ASDAS ≥ 1.3) despite treatment with NSAIDs, or who were unable to take NSAIDs due to contraindications, were included in the study. For all AS patients, the clinical and laboratory assessment was the first time they had been treated with TNF-α inhibitors. Exclusion criteria were any significant comorbidity and the use of any drug other than NSAID or a proton pump inhibitor in the 3 months before the study. Active-stage AS patients received a subcutaneous injection of etanercept biosimilar: Anbainuo (recombinant human tumor necrosis factor-α receptor II: IgG Fc fusion protein for injection, Zhejiang Hisun Pharmaceutica, China) [14] 50 mg weekly for 12 weeks. Peripheral blood lymphocytes were tested with flow cytometry on the baseline and after Anbainuo treatment. Parameters of disease activity, including ASDAS, CRP, and Bath Ankylosing Spondylitis Disease Activity Index (BASDAI) [15] before and after treatment, were also recorded.

### Blood sampling

Blood samples were taken in the laboratory unit of the Department of Rheumatology and Immunology. After peripheral venous blood sampling, 5-mL samples preserved with heparin sodium anticoagulant tubes were used to detect immune cells, and 3-mL samples preserved with blood collection tubes without anticoagulant were used to conduct a CRP test. The samples were stored at room temperature (20–30 °C) for no more than 6 h before handling.

### Lymphocyte immunophenotyping by flow cytometry

Immunophenotyping of peripheral blood lymphocytes was quantified by flow cytometry (BD) according to the manufacturer’s instructions. Whole blood was added to a panel of the following fluorescently labeled antibodies for incubation (for 15 min at room temperature in the dark): CD3-PerCP-Cy5.5, CD4-APC-H7, CD8-BV510, CD127-BV421, CD25 L-PE, CD45RAL-FITC, CCR7-AF647, CD28-PE-Cy7, CD3-APC-H7, CD4-PE-Cy7, CD8-PerCP-Cy5.5, CD183(CXCR3)-Alexa488, CD196 (CCR6)-BV510, CD185 (CXCR5)-Alexa 647, CD194-BV421, CD279(PD-1)-PE for T cell subsets; and CD45-APC-H7, CD19-PerCP-Cy5.5, CD27-BV421, IgD-BB515, IgM-BV510, CD38L-APC, CD24L-PE, and CD21-PE-Cy7 for B cell subsets. Supplementary Table 1 details more information about the categorizations of T/B cell surface markers. After red blood cells were lysed (4 °C in the dark) and centrifuged, the cells were suspended with phosphate-buffered saline and then analyzed on a BD flow cytometry.

### CRP analysis

CRP was tested by immunoturbidimetry in venous blood serum obtained at the same time.

### Statistical analysis

Statistical analysis was carried out using SPSS 23.0 software. The Shapiro-Wilk test was used to test for normality. Reference ranges were calculated using mean ± standard deviation for normally distributed data and median (interquartile range (IQR)) for non-normally distributed data. For data that conformed to a normal distribution, a parameter test was used, and for data that did not conform to a normal distribution, a non-parametric test was used. The difference between the two groups was compared using 2-sided tests for parametric data and the Wilcoxon rank-sum test for non-parametric data. The gender ratio was compared between the control subjects and patients using a chi-square test. Correlation between clinical parameters and the ratio of lymphocyte subtypes was tested using the parametric Pearson correlation coefficient test or the non-parametric Spearman’s rank correlation coefficient test. Statistical significance was considered to exist when *P* < 0.05. A heat map was created using the R package “pheatmap.”

## Results

### Case summary

Supplementary Table 2 compares the characteristics of the AS patients and HCs in two phases: the primary screening phase and the expanded validation phase.

### Primary screening phase

The 28 T lymphocyte and 12 B lymphocyte subset content was analyzed in the AS and HC groups at both the primary screening phase and expanded validation phase. There was a significant change in the percentage of T lymphocytes and B lymphocytes in the AS patients compared to the HCs. The percentage of Th1 cells (CD3+CD4+CXCR3+CCR4−CXCR5−), Tfh1 cells (CD3+CD4+CXCR3+CCR4−CXCR5+), Tc1 cells (CD3+CD8+CXCR3+CCR4−CXCR5−), memory B cells (CD3−CD19+CD27+CD24+CD38+IgD+IgM+), and non-switched B cells (CD3−CD19+CD24−CD27+CD38+IgD+IgM+) were found to be significantly higher in the AS patients. However, the percentage of Th2 cells (CD3+CD4+CXCR3−CCR4+CXCR5−CCR6−), Th17 cells CD3+CD4+CXCR3−CCR4−CXCR5−CCR6+), Tfh2 cells (CD3+ CD4+CXCR3−CCR4+CXCR5+), and B cells (CD3−CD19+) were found to be significantly lower in the AS patients. The statistical results are listed in Supplementary Table 3 (*P* < 0.05). Representative cytofluorometric gating strategy and detailed examples of analyses for the subsets have been represented in supplementary fig. 1.

### Expanded validation phase

**Fig. 1:**
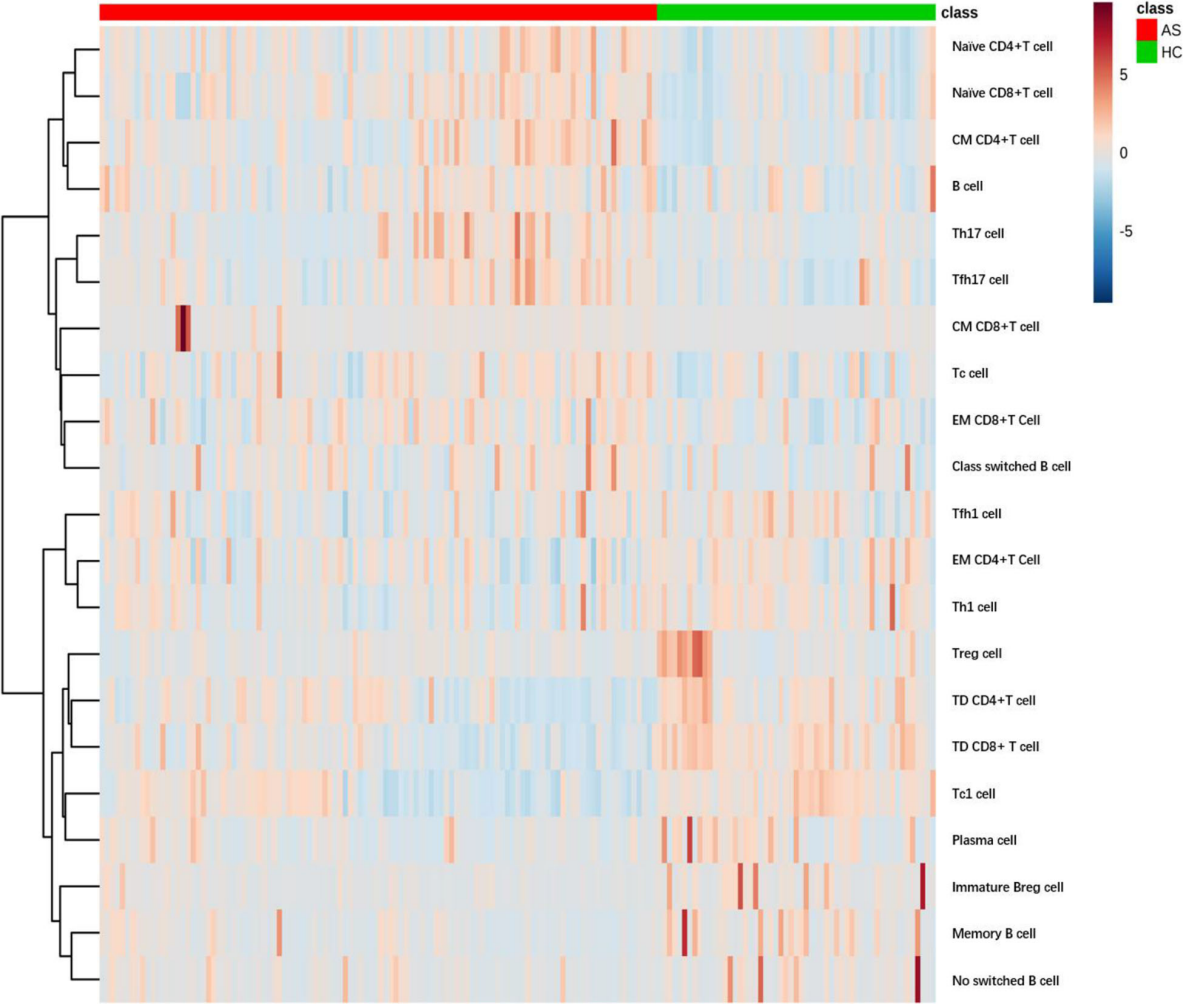
Cluster analyses of immunophenotypic parameters. Each column represents individual AS patient or HC, and the color code in the first line above the graph indicates AS group (red) or HC group (green). The rows represent immune cells that are differentially expressed in AS and HC with a *P* value < 0.05. The magnitude of parameter expression is color-coded with red for a relative increase in expression and blue for a relative decrease in expression. CM CD4+T cell, central memory CD4+T cell; EM CD4+T cell, effector memory CD4+T cell; CM CD8+T cell, central memory CD8+T cell; EM CD8+T cell, effector memory CD8+T cell; Th cell, helper T cell; Tfh cell, follicular helper T cell; Tc cell, cytotoxic T lymphocyte; Treg cell, regulatory T cell; Breg cell, regulatory B cell

Cluster analyses of immunophenotypic parameters that were differentially expressed in the AS patients and HCs at the expanded validation phase are summarized in Fig. 1. The magnitude of parameter expression is color-coded with red for a relative increase in expression and blue for a relative decrease in expression. Strong cluster separation can be observed between AS and HC. Part of immune cells with a relative increase in expression (upper left quadrant and lower right quadrant) clearly separated from others with a relative decrease in expression (upper right quadrant and lower left quadrant). Supplementary Table 4 shows the statistical results of average frequencies and *P* values of a total of 21 immune cells with significant differences between the two groups.

### T lymphocyte

**Fig. 2:**
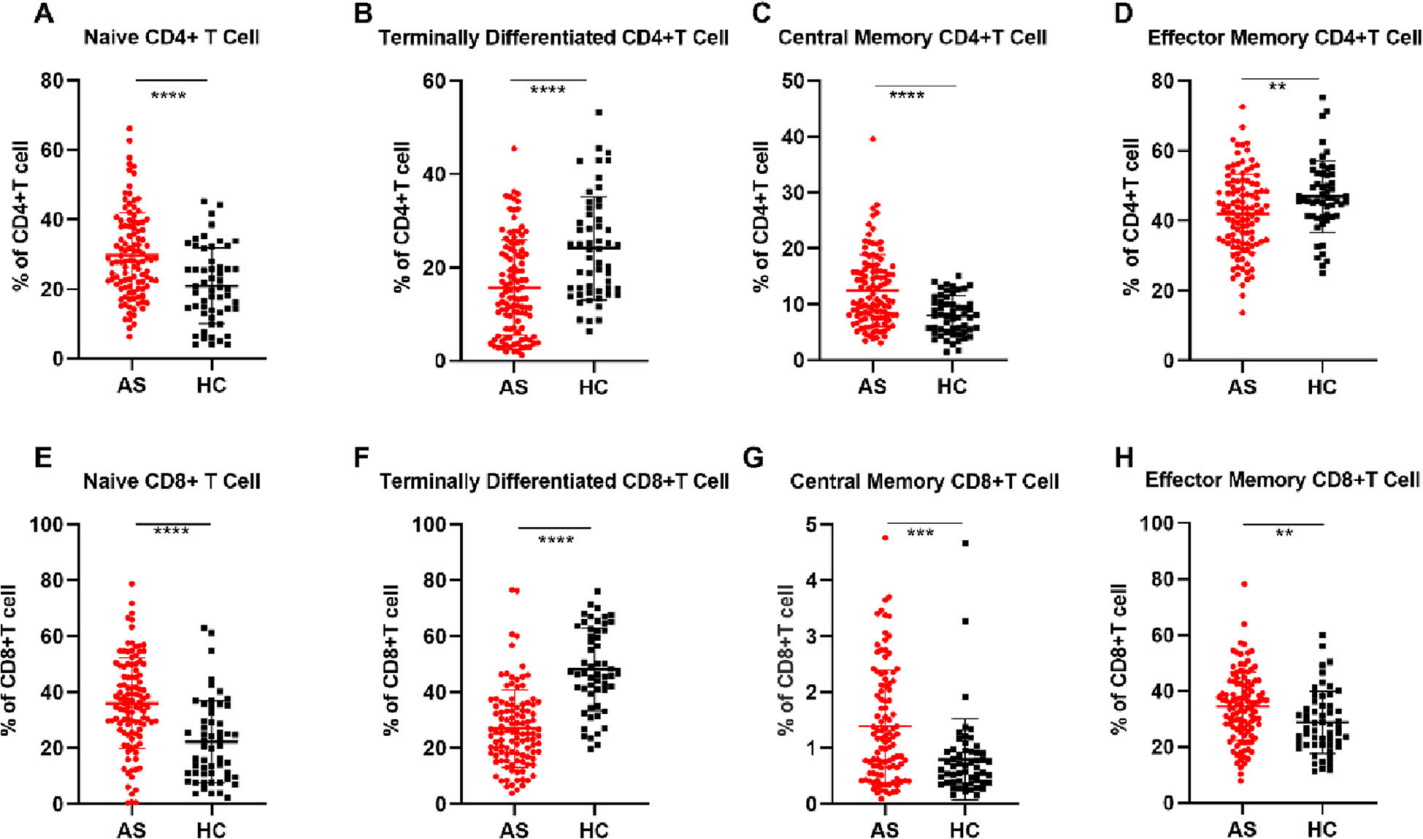
Differences in CD4+ T cells and CD8+ T cells in the AS and HC groups at different stages of differentiation. *P* value summary: *P < 0.05, **P < 0.01, ***P < 0.001, ****P < 0.0001

The percentage of CD4+ T cells at different stages of differentiation were calculated, and significant differences between the AS patients and HCs are shown in Fig. 2. CCR7+ CD4+T cells including naïve CD4+T cells (CD3+CD4+CD45RA+CCR7+, Fig. 2a) and central memory CD4+T cells (CD3+CD4+CD45RA−CCR7+, Fig. 2c) were significantly increased in the AS group, but CCR7− CD4+T cells including terminally differentiated CD4+T cells (CD3+CD4+CD45RA+CCR7−, Fig. 2b), and effector memory CD4+T cells (CD3+CD4+CD45RA−CCR7−, Fig. 2d) were significantly decreased.

As shown in Fig. 2, the percentage of CD8+ T cells at different stages of differentiation was also calculated. Naïve CD8+T cells (CD3+CD8+CD45RA+CCR7+, Fig. 2e), central memory CD8+T cells (CD3+CD8+CD45RA−CCR7+, Fig. 2g), and effector memory CD8+T cells (CD3+CD8+CD45RA−CCR7−, Fig. 2h) were significantly increased in the AS group, but terminally differentiated CD8+T cells (CD3+CD8+CD45RA+CCR7−, Fig. 2f) were significantly decreased.

**Fig. 3:**
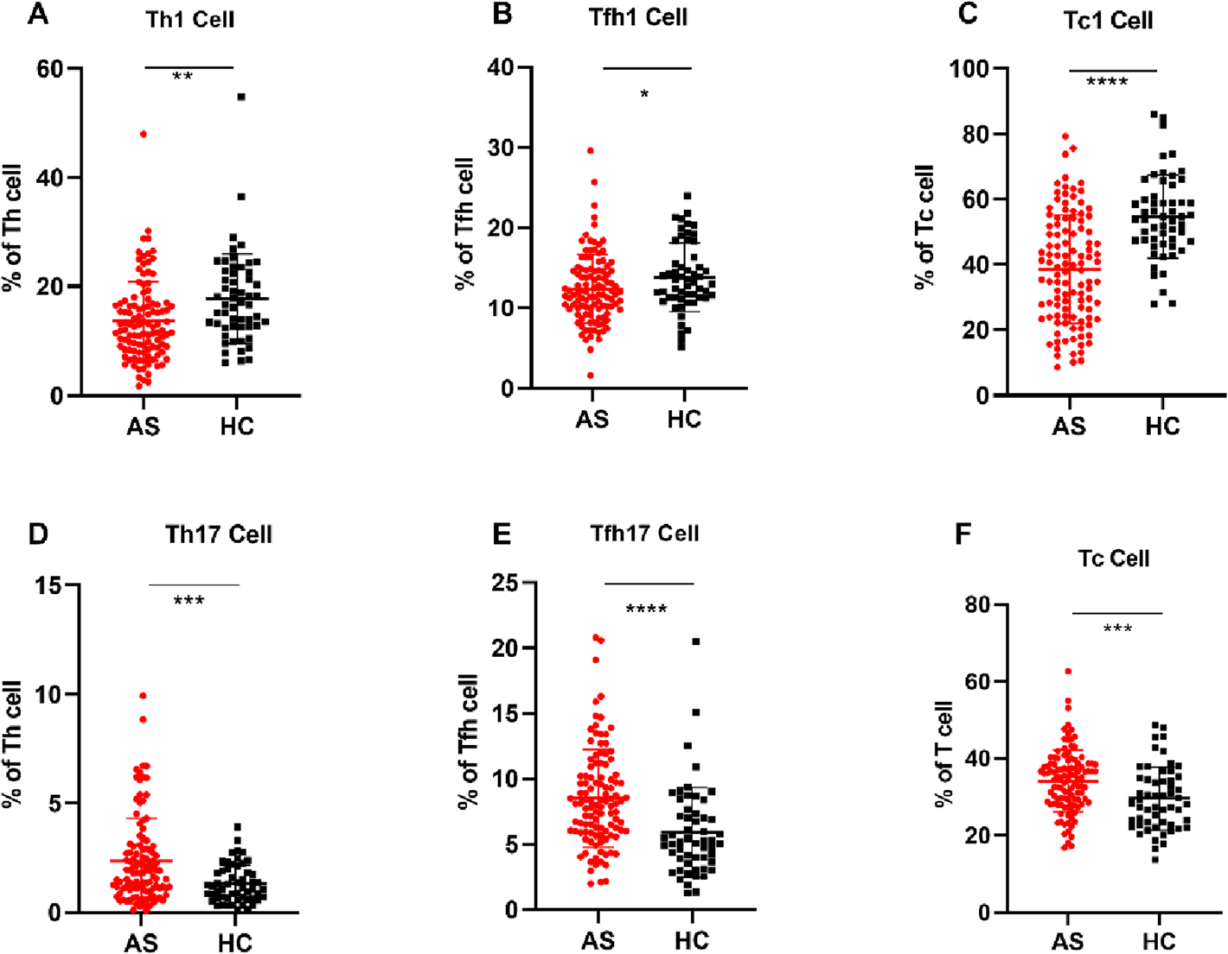
Differences in the percentage of Th cells, Tfh cells, and Tc cells in the AS and HC groups. *P* value summary: *P < 0.05, **P < 0.01, ***P < 0.001, ****P < 0.0001. Th cell, helper T cell; Tc cell, cytotoxic T lymphocyte; Tfh cell, follicular helper T cell

Simultaneously, we tested Th cells (Th1 cells, Th2 cells, Th17 cells), Tc cells (Tc1 cells, Tc2 cells, Tc17 cells), and Tfh cells (Tfh1 cells, Tfh2 cells, Tfh17 cells), and the results with significant differences are shown in Fig. 3. The percentage of CXCR3+T cells, including Th1 cells (CD3+CD4+ CXCR3+ CCR4−CXCR5−, Fig. 3a), Tfh1 cells (CD3+CD4+CXCR3+CCR4−CXCR5+, Fig. 3b), and Tc1 cells (CD3+CD8+CXCR3+ CCR4−CXCR5−, Fig. 3c), was found to be significantly lower in the AS group. However, the percentage of CCR6+ helper T cells, such as Th17 cells (CD3+CD4+CXCR3−CCR4−CXCR5−CCR6+, Fig. 3d) and Tfh17 cells (CD3+CD4+CXCR3−CCR4−CXCR5+CCR6+, Fig. 3e), were found to be significantly higher. The total Tc cells (CD3+CD8+) (Fig. 3f) were also significantly increased in the AS group.

### Regulatory lymphocytes

We compared changes in the ratio in negative regulatory cells, such as Tregs and Bregs, between the AS and HC groups. The percentage of Tregs (CD3+CD4+CD25+CD127−) and immature Bregs (CD3−CD19+CD24+CD27−CD38+IgD+IgM+) was found to be significantly lower in the AS group than in the HC group (*P* < 0.0001). Supplementary Fig. 2 lists the scatter plot results. B10 cells (CD3−CD19+CD24+CD27+CD38−IgD+IgM+) were also shown to be decreased in the AS group, but the difference was not significant.

### B lymphocytes

**Fig. 4:**
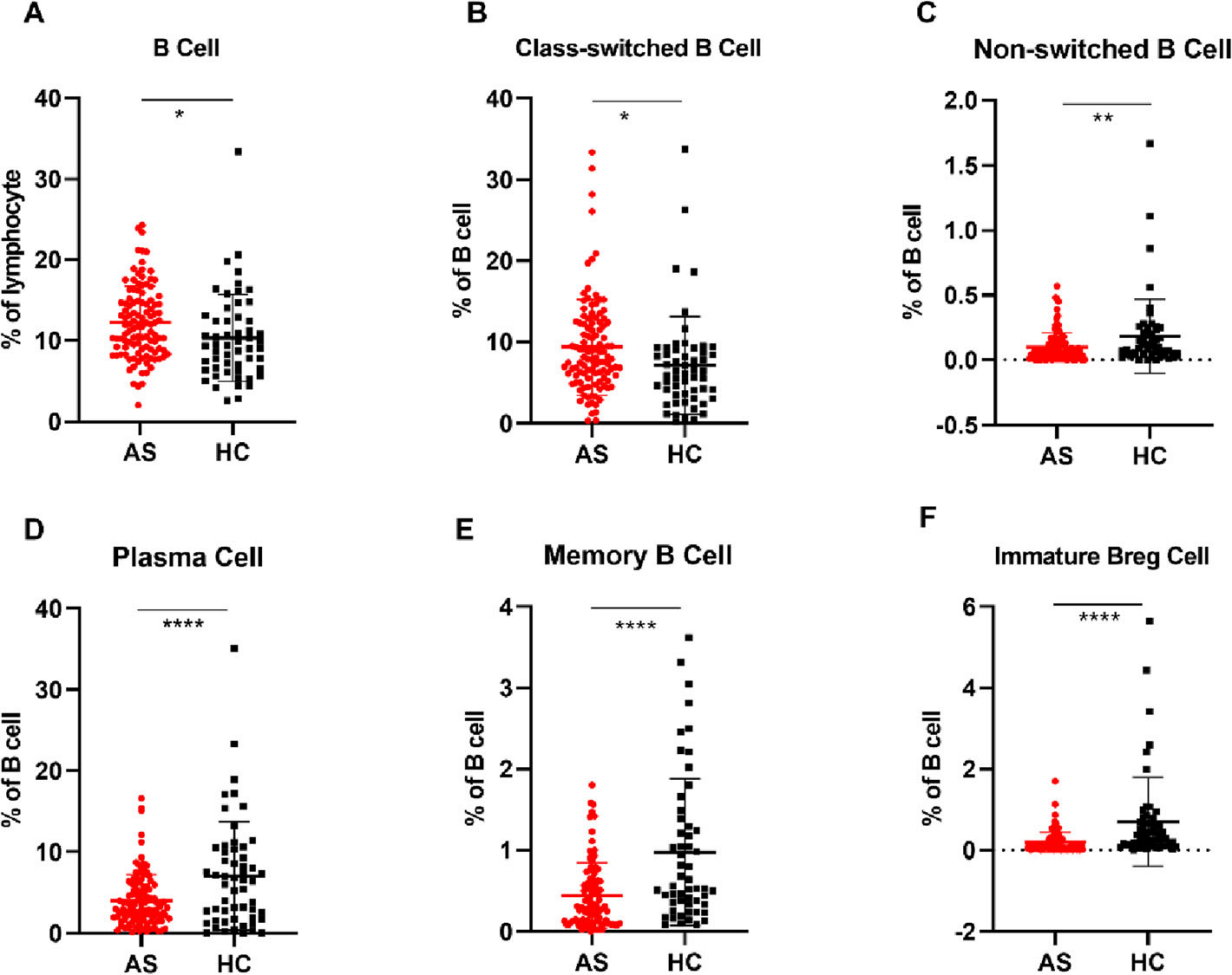
Differences between AS and HCs in B cell percentage. *P* value summary: *P < 0.05, **P < 0.01, ***P < 0.001, ****P < 0.0001

Figure 4 shows the percentage of B cells at different stages of differentiation. The proportion of total B cells (CD3−CD19+, Fig. 4a) and class-switched B cells (CD3−CD19+CD27+CD38−IgD−IgM−, Fig. 4b) were all significantly increased in the AS group. However, antibody-secreting phenotype B cells (CD19+ CD38+) including non-switched B cells (CD3−CD19+CD27+CD38+CD24−IgD+ IgM+, Fig. 4c), plasma cells (CD3−CD19+CD27+CD38+IgD−IgM−, Fig. 4d), memory B cells (CD3−CD19+CD24 +CD27+CD38+IgD+IgM+, Fig. 4e), and immature Bregs (CD3−CD19+IgD+IgM+ CD27−CD38+CD24+, Fig. 4f) were found to be significantly decreased in the AS group compared to the HC group.

### The impact of Anbainuo therapy on lymphocyte subsets in AS

A total of 23 active-phase AS patients were included in this research. The mean age of the patients (M/F:19/4) was 30 years (range, 25–36 years), and mean disease duration was 8 years (range, 4.5–13.00 years) at baseline. Disease activity was indicated by CRP of 12.10 mg/L (range, 2.60–20.90 mg/L), ASDAS of 2.97 ± 1.02, and BASDAI of 4.25 ± 1.37 before treatment. After 12 weeks of Anbainuo therapy, CRP, ASDAS, and BASDAI all decreased significantly after treatment (*P* < 0.05), and the average values were 2.50 mg/L (range, 0.50–8.00 mg/L), 1.37 ± 1.04, and 1.69 ± 1.32, respectively, as shown in Supplementary Table 5.

**Fig. 5:**
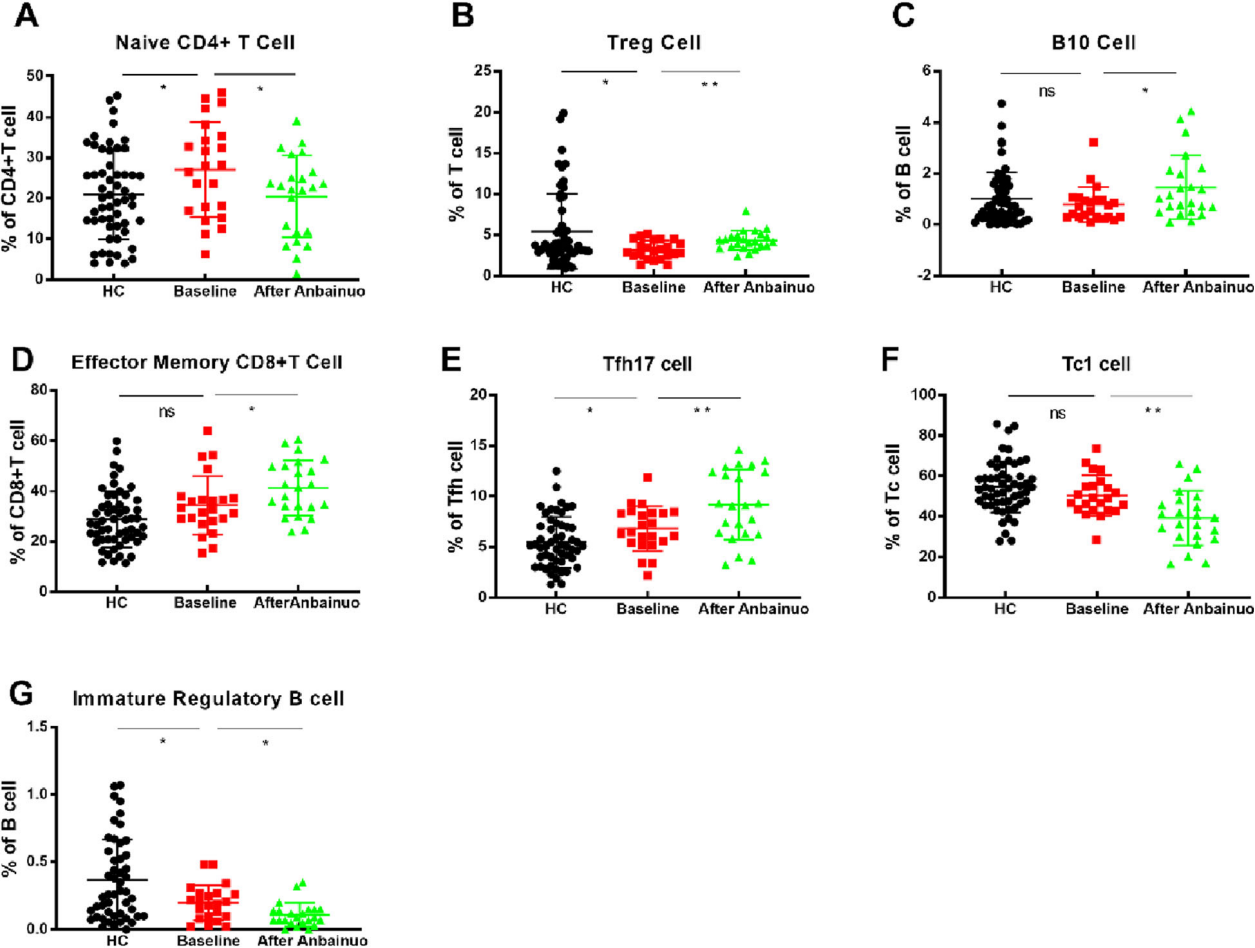
Following Anbainuo treatment, some T cell and B cell subtypes displayed significant changes. We compared in 55 HCs and 23 Anbainuo-treated AS patients. Anbainuo, recombinant human tumor necrosis factor-α receptor II: IgG Fc fusion protein for injection, made in China. *P* value summary: *P < 0.05, **P < 0.01, ***P < 0.001, ****P < 0.0001. Treg cell, regulatory T cell; Tc cell, cytotoxic T lymphocyte; Tfh cell, follicular helper T cell; B10 cell, IL-10 producing regulatory B cell

After 12 weeks of Anbainuo therapy, the amount of some lymphocyte subsets in the peripheral blood of the AS patients changed significantly (Fig. 5). CD4+T cells and CD8+T cells were measured at different stages of differentiation, and comparisons were made between the AS and HC groups. As shown in Fig. 5, naïve CD4+ T cells (CD3+CD4+CD45RA+CCR7+, Fig. 5a) were decreased and effector memory CD8+ T cells (CD3+CD8+CD45RA−CCR7−, Fig. 5d) increased after Anbainuo therapy.

The number of regulatory lymphocytes detected in the blood of the AS patients changed significantly after Anbainuo treatment, with the percentage of Treg cells (CD3+CD4+CD25+CD127−, Fig. 5b) and B10 cells (CD3−CD19+CD24+CD27+ CD38−IgD+IgM+, Fig. 5c) increasing significantly but immature Bregs (CD3−CD19+CD24+ CD27CD38+IgD+IgM+, Fig. 5g) decreasing significantly.

Simultaneously, we measured the number of Th cells (Th1 cells, Th2 cells, Th17 cells), Tc cells (Tc1 cells, Tc2 cells, and Tc17 cells), and Tfh cells (Tfh1 cells, Tfh2 cells, and Tfh17 cells) before and after Anbainuo therapy. As shown in Fig. 5, the proportion of Tc1 cells (CD3+CD8+CXCR3+CCR4−CXCR5−, Fig. 5e) decreased, and the proportion of Tfh17 cells (CD3+CD4+CXCR3−CCR4−CXCR5+ CCR6+, Fig. 5f) increased after treatment.

However, apart from immature Bregs and B10 cells, the proportion of various B cell subtypes did not change significantly after treatment with Anbainuo.

### Correlation between immune cells and disease activity

In order to understand whether disease activity of AS patients is related to immune cell imbalance, we analyzed the correlation between disease activity indicators (CRP and ASDAS) and frequency of immune cells. But only the frequency of Tc1 cells (CD3+CD8+CXCR3+CCR4−CXCR5−) was found to be negatively correlated with CRP level (*r* = − 0.182, *P* = 0.041).

To understand the correlation between changes in disease status (including CRP, BASDAI, and ASDAS) and changes in lymphocyte frequency after Anbainuo therapy, Spearman’s rank correlation analyses showed that the decrease in CRP was positively correlated with the increase in the frequency of Tregs (CD3+CD4+CD25+CD127−) following Anbainuo therapy for 12 weeks (*r* = 0.489, *P* = 0.018).

## Discussion

As we know, the onset of AS suffers from the relationship between the host genetics, the intestinal microbiome, and the immune response [16]. AS has long been associated with inheritance of the HLA allele B27 [1], and the pathogenic role of HLAB27 remains unclear despite intensive research. The arthritogenic peptide theory proposes that HLAB27 plays a central pathogenic role in the presentation of joint-specific peptides to CD8+ cytotoxic T cells. Specific self or environmental peptides are proposed to bind to and be presented by HLA-B27, to activate CD8+ cells. Another major theory for the pathogenesis of HLA-B27 in AS revolves around the ability of HLA-B27 to aberrantly fold to form homodimers [17]. Circulating CD4+ T cells, expressing the killer cell immunoglobulin receptor (KIR3DL2) after activation, recognize HLA-B27 homodimers, and this recognition is associated with the secretion of large amounts of inflammatory cytokines including high levels of IL-17A, and these cells are polarized toward a Th17 phenotype [18]. Our research is basically consistent with the above immunological concepts in the pathogenesis of AS. We found that the proportion of naïve CD4+/CD8+T cells and memory CD4+/CD8+T cells had increased while terminally differentiated CD4/CD8+T cells had decreased, which just confirmed the role of activation of CD4+/CD8+ T cells in the classic theory of AS pathogenesis.

One of the most specific pathologic features of AS is inflammation at the enthesis [19]. Recent genetic and immunological research has highlighted a key role for IL-17A/IL-23 cytokine dysregulation of the Th17 immune pathway in AS [1]. IL-23 signaling through the IL-23 receptor (IL-23R) on CD4+ Th cells is required for the differentiation and expansion of Th17 cells. Mechanical stress and/or infectious stress resulting in overexpression of the IL-17A/IL23 axis with activation of resident entheseal cells that can respond to IL-23 including CD4+T cells, CD8+ T cells, γδT cells, and other innate immune cells. This leads to production of IL-17A, IL-22, TNF-α, and other cytokines that mediate spinal and peripheral inflammation directly or through tissue-resident effector cells [20]. A perpetual activation of both the innate and the adaptive immune systems, in particular of the IL-23/IL-17 axis and of Th1 effector T cell lineage with the overproduction of TNF-α, is considered to be key steps in the pathogenesis of AS. We also found that the proportion of Th17 cells increased while the proportion of Th1 cells decreased, confirming the key role of IL-17A/IL-23 signaling pathway in the pathogenesis of AS.

The inflammatory effects seen throughout AS are associated with immune imbalance. Previous studies have found changes in the immune cells of patients with AS [21–23]. Our study, which included an extensively validated large sample size, showed that active AS causes abnormalities in the proportion of lymphocyte subsets and that help us to indirectly understand the differentiation status, degree of failure, and cell activity of various cell subsets. Our results, which showed changes in the frequency of Th cells, Tfh cells, Tc cells, and Treg cells in patients with AS were consistent with the findings of past studies [3, 8, 22]. We also made a discovery regarding abnormal changes in CD4+T cells and CD8+T cells at different stages of differentiation of AS, which further suggests that AS patients have an irregular proportion of T cells at different stages of differentiation. Some previous studies based on small sample sizes found only abnormal changes in CD4+T cell subsets at different stages of differentiation but not in CD8+T cell subsets [24].

TNF-α induces the production of multiple inflammatory factors and has an impact on the differentiation and activation of various T cell subtypes, and therefore, it has a significant role in shaping the adaptive immune system [25]. Our results showed that Anbainuo injections could address partial immune imbalance in AS patients by affecting differentiation and activation of immune cell subtypes, including naïve CD4+ T cells, Tregs, and B10 cells. Among these, the decreased proportion of naïve CD4+ T cells after Anbainuo treatment was consistent with the findings of past research [26]. Our observation that naïve CD4+ T cells decreased but effector memory CD8+ T cells increased after Anbainuo treatment is in line with our expectations, because there are many reasonable explanations for this result. One possible explanation is that migration of T cell precursors from the thymus or release of naïve T cells from the secondary lymphoid organs may have been impaired, but differentiation of memory T cells from naïve cells may have accelerated. This could be one of the mechanisms by which Anbainuo regulates immunity and plays an anti-inflammatory role.

However, we think it makes more sense that Anbainuo treatment for AS can increase the proportion of negative regulatory cells such as Tregs and Bregs in inflammation that are significantly lower than normal before treatment. We have described the decreased frequency of Tregs, immature Bregs, and B10 cells, in AS patients who had previously received no biological treatment agents (we found that B10 cells did not display a significant change between AS and HCs). This may suggest that the lack of negative regulatory cells in inflammation may contribute to the pathogenesis of AS. The parallel compensatory increase seen in Tregs and B10 cells after Anbainuo treatment for 12 weeks suggests that TNF-α inhibitor may assist with the recovery of patients’ immune tolerance.

Tregs are a small subset of CD4+ T cells that play a pivotal role in the maintenance of immunological tolerance and prevention of autoimmunity [27]. It has been reported that decreases in Treg number and function lead to abnormal immune responses toward self-antigens, thus resulting in rheumatic diseases including AS. Recent report showed that the mean fluorescence intensity (MFI) of FOXP3 in circulating Treg cells was significantly decreased in active AS patients, and Treg cells could not effectively inhibit the proliferation of naïve T cell [6]. In addition, active AS patients harbor Treg cells that are defective in using IL-2, have relatively little STAT5 phosphorylation, and have higher CpG methylation levels in CNS2 region of the foxp3 gene [6]. It suggested that the functional defects of Treg cells are present and play important roles in AS. Although higher values of CRP, together with the presence of HLA-B27, have been reported to be useful baseline predictors for a successful anti-TNF-α therapy response in AS, the robustness, sensitivity, and specificity fail if applied to individual patients. Given that Treg cells are powerful immunosuppressive agents, manipulating Treg cells is a new therapeutic strategy for treating autoimmune diseases. An increase in Tregs during long-term anti-TNF therapy for AS has also been noted [26]. Our research further reveals a positive correlation between the reduction in CRP value and a higher frequency of Tregs. We propose a hypothesis that low Tregs frequency at the initiation of anti-TNF therapy may be a good predictor of treatment response to anti-TNF treatment in AS. Although our findings are promising, a further validation of Tregs as a potential biomarker for TNF responsiveness is necessary in an independent cohort of AS and other rheumatic and gastrointestinal diseases where anti-TNF blockers are successfully administered.

In humans, plasmablasts, immature B cells, B10 cells, B regulatory 1 (Br1) cells, and Granzyme B (GrB)+ B cells comprise the identified Breg subsets [28]. Bregs cells contain a high proportion of IL-10-producing Bregs cells, named B10 cells [29], which, via the production of IL-10, suppress TNF-α production by monocytes. Human regulatory plasmablasts are suggested to drive from immature B cells and act through IL-10 production [30, 31]. This may explain our observation that immature Bregs decreased but B10 cells increased after Anbainuo treatment. However, another small sample size study about Bregs led to the opposite conclusion [7]. IL-10 secreted by B10 cells plays a role in the development of intestinal immunity [32]. Environmental factors, which may include the microbiome and infections, probably also play a significant role in AS pathogenesis [1]. Bowel inflammation and AS are frequently clinically associated [33]. Our finding that B10 cells increased after Anbainuo treatment may provide further support to the hypothesis that AS is caused by interactions between the host gut immune system and the gut microbiome. IL-10 secreted by B10 cells could be used as potential indicators for the therapeutic efficacy of TNF-α inhibitors in AS with bowel inflammation.

However, Anbainuo is not able to regulate all of the immune imbalances attributed to AS. Our results show that the proportion of effector memory CD8+ T cells and Tfh17 cells, which was significantly higher in AS patients (110 AS patients vs. 55 HCs), and the proportion of Tc1 cells, which was significantly lower in AS patients (110 AS patients vs. 55 HCs), have not been rescued after treatment. Initially, we found the frequency of Tc1 cells to be significantly decreased and to be negatively associated with CRP levels. Although we have to admit that the actual significance of the correlation coefficient between CRP and Tc1 is indeed limited, we still expected that after treatment with TNF inhibitors, the frequency of Tc1 cells will increase because the inflammatory process has been put to quiescence clinically as reflected by routine laboratory inflammatory marker (CRP) and disease activity index (ASDAS and BASDAI). However, after Anbainuo treatment, although the disease activity of AS patients improved, the frequency of Tc1 cells was still lower than the baseline, and at the same time, the results of correlation analysis showed that neither BASDAI nor ASDAS had anything to do with the changes of immune cell phenotype. The present measurements confirm our previous hypothesis that the major mechanism of action of anti-TNF therapy is to obtain inhibition of the inflammatory process, not the restoration of the activated immune system from active disease to a state similar to healthy individuals, in terms of the immune cell subset distribution. It seems that the generation of differentiated CD8+ T cells (such as effector memory CD8+T cell and Tc1 cell in the result) remains an ongoing process despite TNF blockers, and the T cell repertoire necessary for an effective antitumor or antimicrobial defense is not compromised. Now that some immunological abnormality cannot be rescued despite the effects of Anbainuo, further study is required to clarify whether the abnormalities of the CD8+ T cells in the frequency or functions are related to resistance that could contribute to relapse after treatment. The value of these immune cells as new potential targets for AS therapy is also worthy of attention in future research.

In the past, there have been a small number of reports on the abnormal distribution of B cells in the peripheral blood of AS patients [23]. However, the distribution of different subtypes of B cells or different stages of differentiation has not yet been fully revealed. Our study found that AS patients experienced a significant increase in the total of B cells and class-switched B cells but a significant reduction in the proportion of antibody-secreting subtype B cells during the B cell effector phase. At the same time, no significant difference was observed in terms of autoimmune-related changes in the AS patients compared with the HCs. This shows that AS is better defined as an autoinflammatory disease than an autoimmune disease. In the future, potential immune regulation against B cells is likely to benefit patients with AS.

This study has a few weak points. It was found that the average onset age of the first stage (primary screening phase) is lower than general AS population [34]. That may be related to the limited sample size of the first stage without artificial screening for demographic characteristics. That may cause some bias especially the result of immune imbalance associated with the course of the disease. However, the onset age of the second stage (expanded validation phase) (22.7 ± 7.85) is consistent with the general AS population. Here we truthfully report the true statistics and plan to further expand the verification study in the future.

## Conclusion

Our prospective study, which had a large sample size, found active-stage AS patients to have an immunity imbalance of frequency involving multiple immune cells. The pathogenesis of AS sees not only an increase in effector immune cells but also a decrease in negative regulatory immune cells. TNF-α inhibitor Anbainuo can not only help to inhibit disease activity and partial immune cell imbalance of frequency in AS patients by affecting the differentiation and activation of immune cell subtypes of CD4+T cells, but it can also increase the number of negative regulatory cells in inflammation. While Anbainuo therapy improved the overall condition of patients, cell prevalence abnormalities did not disappear and CD8+T cells have not been rescued after treatment. The immune cells that cannot be improved in the treatment of TNF-α inhibitor need to be further investigated in future research.

## Supplementary information

**Additional file 1: Table S1.** The 28 T lymphocyte and 12 B lymphocyte subsets [35]. **Table S2.** Characteristics of AS patients and HCs. **Table S3.** Differences in lymphocyte frequencies between AS patients and HCs at the primary screening phase. **Figure S1**. Representative gating strategy to identify T lymphocyte (A-N) and B lymphocyte (O-X) subsets. CM: Central Memory, EM: Effector Memory, Th cell: Helper T cell, Tfh cell: Follicular helper T cell, Tc cell: Cytotoxic T cell, EMRA cells: terminal differentiated effector memory cells, Treg cell: Regulatory T cell, Breg cell: Regulatory B cell. **Table S4.** Differences in lymphocyte frequencies between the AS patients and HCs at the expanded validation phase. Figure S2. The proportion of regulatory lymphocytes is shown for both the AS and HC groups. *P*-value summary: (*, *P* < 0.05) (**, *P* < 0.01) (***, *P* < 0.001) (****, *P* < 0.0001). Treg cell: Regulatory T cell, Breg cell: Regulatory B cell. **Table S5.** Demographic and disease characteristics of AS patients treated with Anbainuo.

## Data Availability

The datasets used and analyzed during the current study are available from the corresponding author on reasonable request.
