# Supplementary material for: TNF-α inhibitor therapy can improve the immune imbalance of CD4+ T cells and negative regulatory cells but not CD8+ T cells in ankylosing spondylitis
Source: Arthritis Res Ther. 2020 Jun 19;22:149. doi: 10.1186/s13075-020-02226-8 (PMC7304211; doi:10.1186/s13075-020-02226-8)
Supplement: Supplementary file 1 — Additional file 1: Table S1. The 28 T lymphocyte and 12 B lymphocyte subsets [35]. Table S2. Characteristics of AS patients and HCs. Table S3. Differences in lymphocyte frequencies between AS patients and HCs at the primary screening phase. Figure S1. Representative gating strategy to identify T lymphocyte (A-N) and B lymphocyte (O-X) subsets. CM: Central Memory, EM: Effector Memory, Th cell: Helper T cell, Tfh cell: Follicular helper T cell, Tc cell: Cytotoxic T cell, EMRA cells: terminal differentiated effector memory cells, Treg cell: Regulatory T cell, Breg cell: Regulatory B cell. Table S4. Differences in lymphocyte frequencies between the AS patients and HCs at the expanded validation phase. Figure S2. The proportion of regulatory lymphocytes is shown for both the AS and HC groups. P-value summary: (*, P < 0.05) (**, P < 0.01) (***, P < 0.001) (****, P < 0.0001). Treg cell: Regulatory T cell, Breg cell: Regulatory B cell. Table S5. Demographic and disease characteristics of AS patients treated with Anbainuo. [file 13075_2020_2226_MOESM1_ESM.docx]

***Supplementary Table 1. The 28 T lymphocyte and 12 B lymphocyte subsets***[17]

| Lymphocyte subsets | Phenotype |
| --- | --- |
| T cell | CD3+ |
| Th cell | CD3+CD4+ |
| Tc cell | CD3+CD8+ |
| DP T cell | CD3+CD4+CD8+ |
| Naïve CD4+T cell | CD3+CD4+CD45RA+CCR7+ |
| Terminally Differentiated CD4+T cell | CD3+CD4+CD45RA+CCR7- |
| Central Memory CD4+T cell | CD3+CD4+CD45RA-CCR7+ |
| Effector Memory CD4+T cell | CD3+CD4+CD45RA-CCR7- |
| Exhausted CD4+T cell | CD3+CD4+CD28- |
| Functional CD4+T cell | CD3+CD4+CD28+ |
| Treg cell | CD3+CD4+CD25+CD127- |
| Naïve CD8+T cell | CD3+CD8+CCR7+CD45RA+ |
| Terminally Differentiated CD8+T cell | CD3+CD8+CCR7-CD45RA+ |
| Central Memory CD8+T cell | CD3+CD8+CCR7+CD45RA- |
| Effector Memory CD8+T cell | CD3+CD8+CCR7-CD45RA- |
| Exhausted CD8+T cell | CD3+CD8+CD28- |
| Tfh cell | CD3+CD4+CXCR5+ |
| Th1 cell | CD3+CD4+CXCR5-CXCR3+CCR4- |
| Th2 cell | CD3+CD4+CXCR5-CXCR3-CCR4+ |
| Th17 cell | CD3+CD4+CXCR5-CXCR3-CCR4-CCR6+ |
| Tfh1 cell | CD3+CD4+CXCR5+CXCR3+CCR4- |
| Tfh2 cell | CD3+CD4+CXCR5+CXCR3-CCR4+ |
| Tfh17 cell | CD3+CD4+CXCR5+CXCR3-CCR4-CCR6+ |
| Tc1 cell | CD3+CD8+CXCR5-CXCR3+CCR4- |
| Tc2 cell | CD3+CD8+CXCR5-CXCR3-CCR4+ |
| Tc17 cell | CD3+CD8+CXCR5-CXCR3-CCR4-CCR6+ |
| Peripheral Th cell | CD3+CD4+CXCR5-PD-1+ |
| Activated Tfh cell | CD3+CD4+CXCR5+PD-1+ |
| B cell | CD3-CD19+ |
| Naïve B cell | CD3-CD19+CD27-IgD+ |
| MZ B cell | CD3-CD19+CD27+IgD+ |
| CD21low B cell | CD3-CD19+IgD+CD27- CD38lowCD21low |
| Pre-Naïve B cell | CD3-CD19+IgM-IgD-CD27-CD38+ |
| Plasma cell | CD3-CD19+IgD-IgM-CD27+CD38+ |
| Class-switched B cell | CD3-CD19+IgD-IgM-CD27+CD38- |
| B10 cell | CD3-CD19+IgD+IgM+CD27+CD38-CD24+ |
| Memory B cell | CD3-CD19+IgD+IgM+CD27+CD38+CD24+ |
| Non-switched B cell | CD3-CD19+IgD+IgM+CD27+CD38+CD24- |
| Immature Breg cell | CD3-CD19+IgD+IgM+CD27-CD38+CD24+ |
| Transitional B cell | CD3-CD19+IgM+IgD-CD27-CD38+CD24+ |

*Th cell: Helper T cell, Tc cell: Cytotoxic T lymphocyte, DP T cell: Double Positive T cell, Treg cell: Regulatory T cell, Tfh cell: Follicular helper T cell, Breg cell: Regulatory B cell.* *CD183:CXCR3,* *CD185:CXCR5,* *CD194:CCR4,* *CD196:CCR6, CD279:PD1.*

***Supplementary Table 2. Characteristics of AS patients and HCs.***

| Parameters | Primary Screening Phase | | | Expanded Validation Phase | | |
| --- | --- | --- | --- | --- | --- | --- |
|  | AS (n=67) | HC (n=50) | P | AS (n=110) | HC (n=55) | P |
| Age (years) | 33 (26-41) | 31.5 (28-38) | 0.976 | 31.5 (25-37) | 30 (27-37) | 0.402 |
| Sex ratio (% male) | 80.60 | 60 | 0.184 | 73.64 | 63.64 | 0.185 |
| D D (years) | 16.75 (11.5-23.75) | - | - | 8 (3-14) | - | - |
| CRP (mg/L) | 10.25 (4.18-26.51) | - | - | 8.50(2.30-18.10) | - | - |
| ASDAS-CRP | 2.89±1.13 | - | - | 2.53±0.97 | - | - |

*Results are displayed as mean ± standard deviation for normally distributed data and median (IQR) for non-normally distributed data. AS ankylosing spondylitis. HC, healthy control. DD disease duration. CRP: C-reactive protein. ASDAS: Ankylosing Spondylitis Disease Activity Score.*

***Supplementary Table 3. Differences in lymphocyte frequencies between AS patients and HCs at the primary screening phase.***

| Subtype | Frequency (%) | | P |
| --- | --- | --- | --- |
|  | AS (n=67) | HC (n=50) |  |
| Th1 cell | 14.06±6.57 | 18.08±8.5 | 0.01 |
| Th2 cell | 14.4 (10.9-18.3) | 12.8 (9.35-15.8) | 0.02 |
| Th17 cell | 1.55 (0.8-2.8) | 1.06 (0.65-1.85) | 0.01 |
| Tfh1 cell | 11.5 (9.57-13.6) | 13.1 (11.28-15.7) | 0.02 |
| Tfh2 cell | 37.75±6.96 | 34.47±7.48 | 0.02 |
| Tc1 cell | 40.62±18.4 | 54.362±12.4 | **** |
| B cell | 12.86±5.53 | 9.92±4.36 | **** |
| Memory B cell | 0.25 (0.13-0.79) | 0.6 (0.35-1.42) | **** |
| Non-switched B cell | 0.05 (0-0.13) | 0.1 (0.05-0.22) | **** |

*Results are displayed as mean ± standard deviation for normally distributed data and median (IQR) for non-normally distributed data. Th cell: Helper T cell. Tfh cell: Follicular helper T cell. Tc cell: Cytotoxic T lymphocyte. (**, P<0.01)*

**A B C**


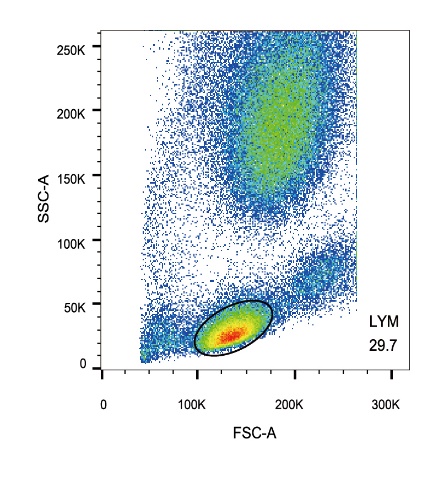

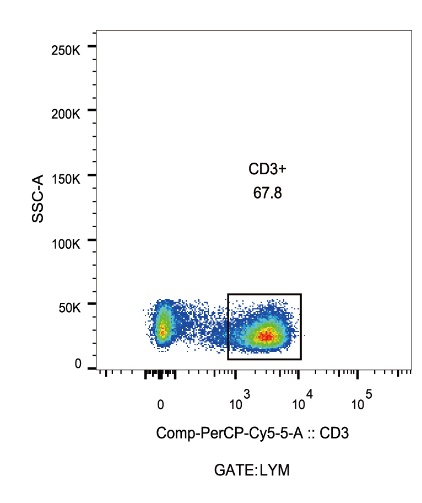

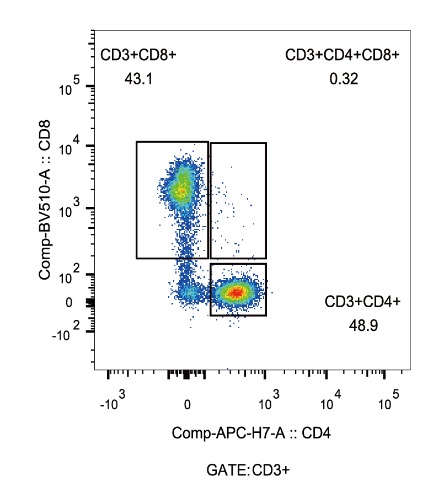


**D E F**


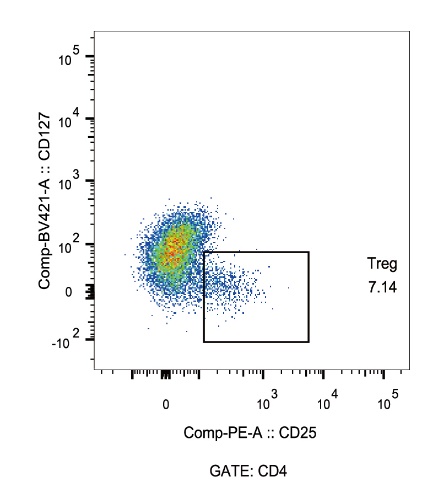

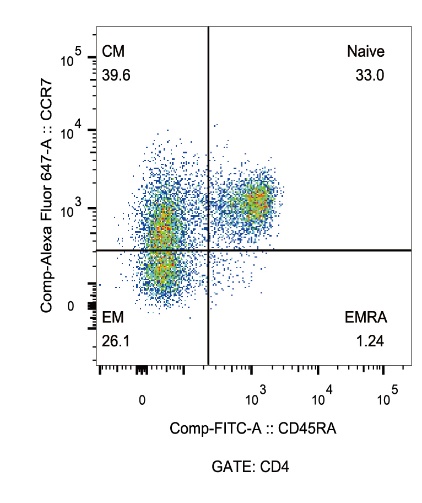

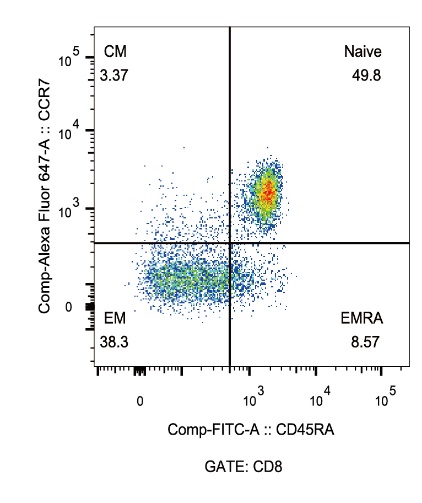


**G H I**


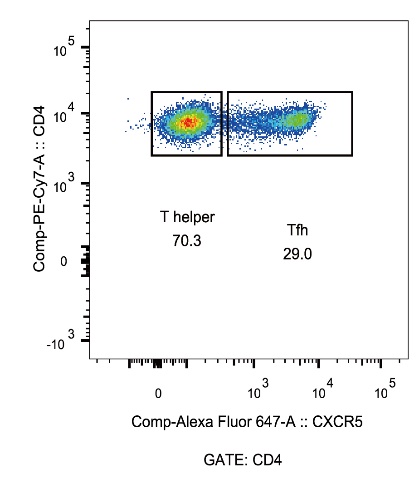

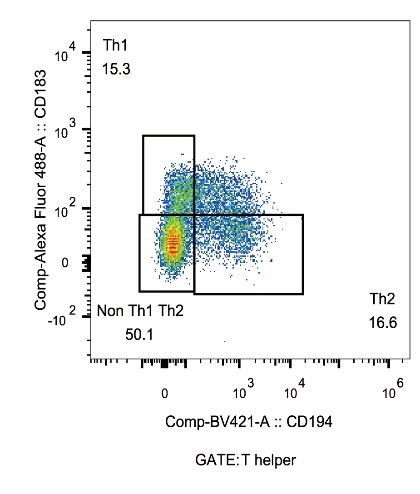

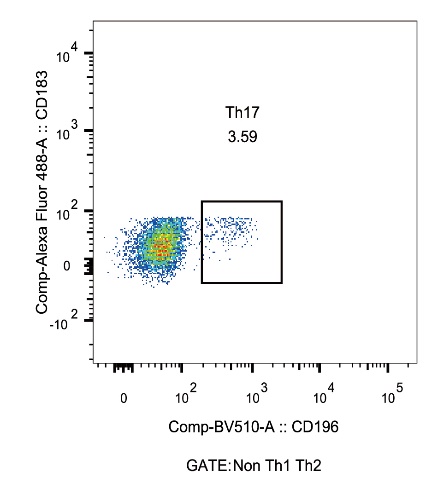


**J K L**


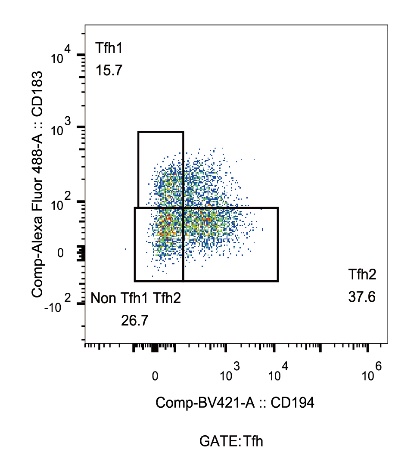

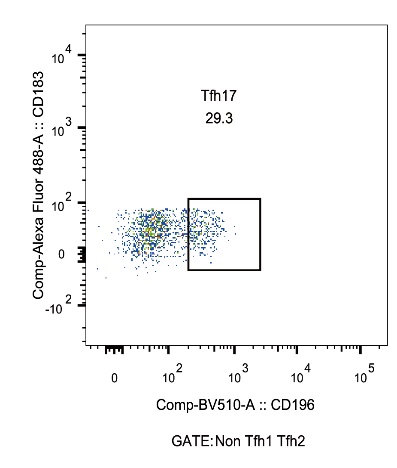

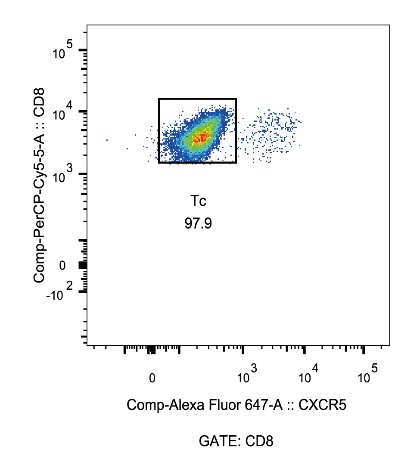


**M N O**


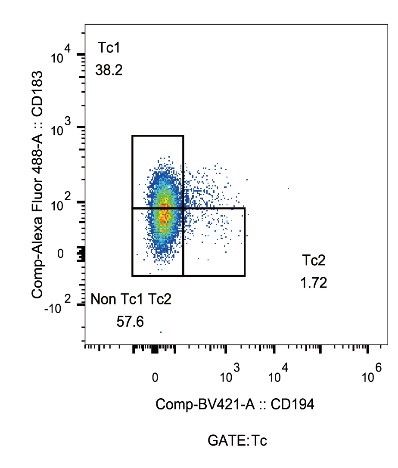

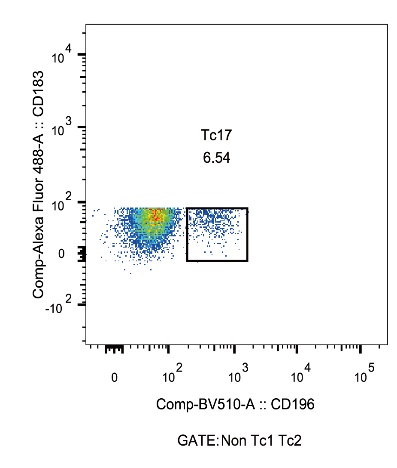

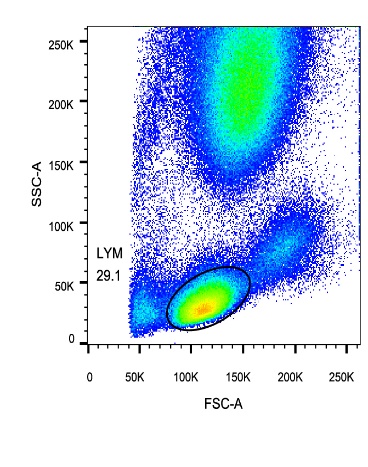


**P Q R**


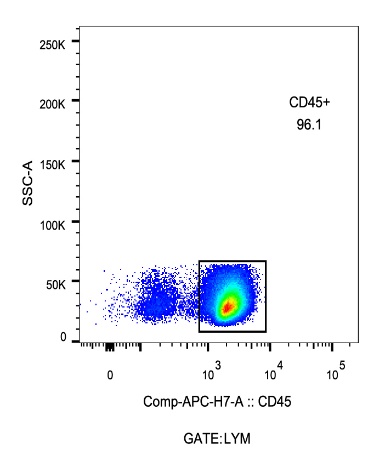

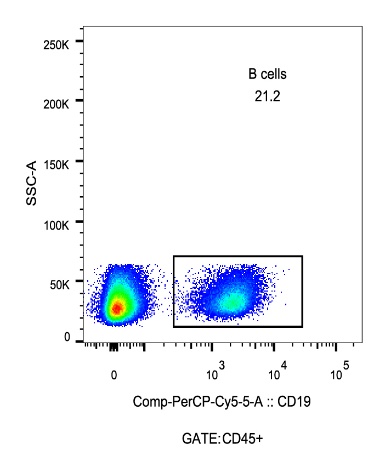

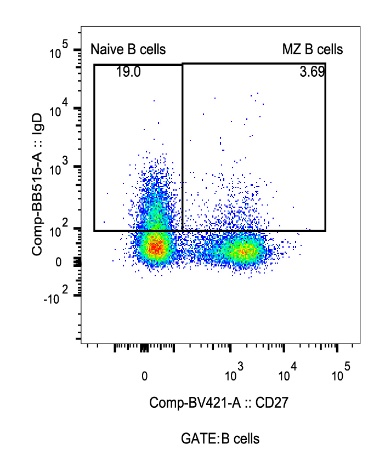


**S T U**


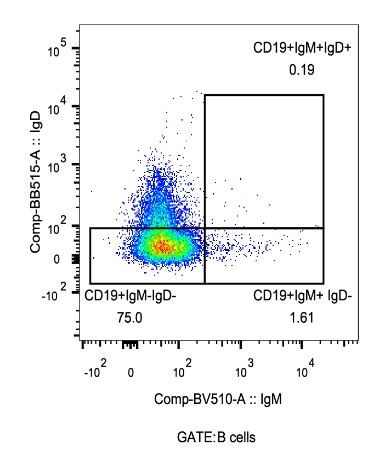

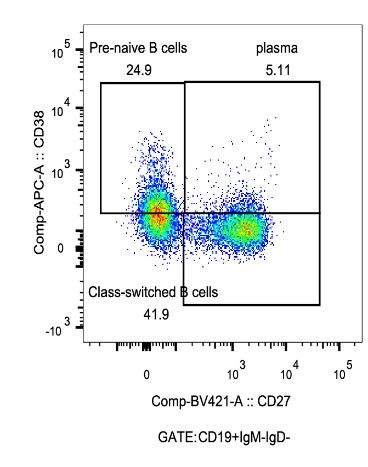

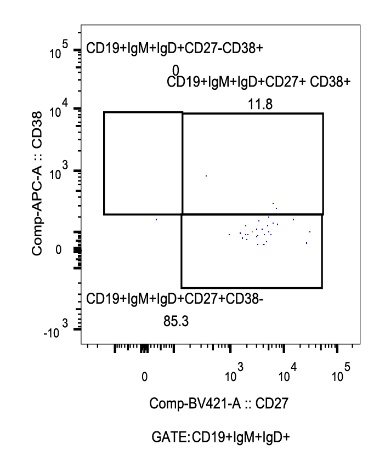


**V W X**


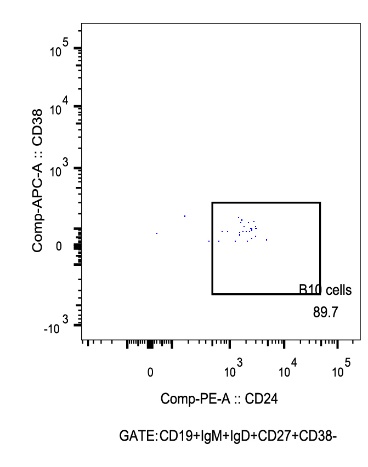

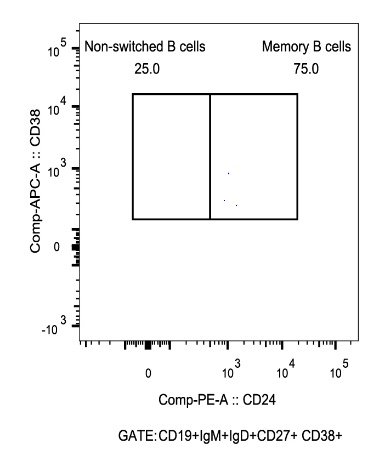

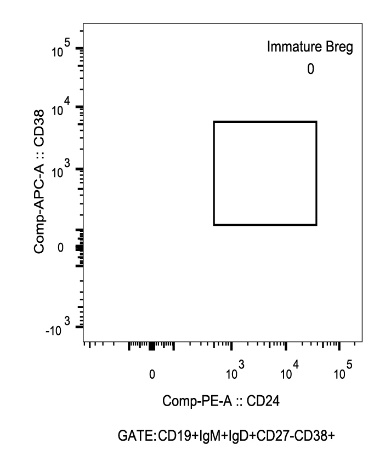


***Supplementary Fig 1. Representative gating strategy to identify T lymphocyte (A-N) and B lymphocyte (O-X) subsets.*** *CM: Central Memory, EM: Effector Memory, Th cell: Helper T cell, Tfh cell: Follicular helper T cell, Tc cell: Cytotoxic T cell, EMRA cells: terminal diﬀerentiated eﬀector memory cells, Treg cell: Regulatory T cell, Breg cell: Regulatory B cell.*

***Supplementary Table 4. Differences in lymphocyte frequencies between the AS patients and HCs at the expanded validation phase.***

| Subtype | Frequency (%) | | P-value |
| --- | --- | --- | --- |
|  | AS (n=110) | HC (n=55) |  |
| Naïve CD4+T cell | 29.77±12.33 | 20.95±10.87 | **** |
| Terminally Differentiated CD4+T cell | 13.5(6.58-23.6) | 23.8(15-31.3) | **** |
| Central Memory CD4+T cell | 10.7(7.62-16) | 7.96(5.07-10.4) | **** |
| Effector Memory CD4+T cell | 42.07±11.24 | 46.90±10.28 | **** |
| Naïve CD8+T cell | 35.75(26.28-48.2) | 20.5(10.9-32.6) | **** |
| Terminally Differentiated CD8+T cell | 27.00±13.90 | 48.15±14.78 | **** |
| Central Memory CD8+T cell | 1.21(0.63-2.26) | 0.66(0.38-0.94) | **** |
| Effector Memory CD8+T cell | 34.58±12.29 | 28.81±11.15 | **** |
| Th1 cell | 12.3(8.42-16.65) | 16.6(12.7-22.6) | **** |
| Th17 cell | 1.82(1.05-2.97) | 1.08(0.66-1.89) | **** |
| Tfh1 cell | 12.40±4.28 | 13.85±4.25 | 0.04 |
| Tfh17 cell | 8.1(5.97-10.23) | 5.13(3.66-7.33) | **** |
| Tc cell | 34.12±8.01 | 29.55±8.23 | **** |
| Tc1 cell | 38.56±16.65 | 54.63±12.72 | **** |
| Treg cell | 3.54±1.21 | 5.45±4.58 | **** |
| B cell | 11.8(8.91-14.93) | 9.43(6.47-12.9) | **** |
| Class-switched B cell | 7.9(5.63-12.3) | 6.1(3.49-8.97) | **** |
| Non-switched B cell | 0.06(0.03-0.13) | 0.09(0.04-0.22) | 0.03 |
| Plasma cell | 3.1(1.82-5.50) | 6.45(1.74-10.3) | **** |
| Memory B cell | 0.31(0.13-0.62) | 0.56(0.33-1.39) | **** |
| Immature Breg cell | 0.11(0.04-0.27) | 0.35(0.12-0.68) | **** |

*Results are displayed as mean ± standard deviation for normal distribution data and median (IQR) for non-normally distributed data.Th cell: Helper T cell. Tfh cell: Follicular helper T cell. Tc cell: Cytotoxic T lymphocyte. Treg cell: Regulatory T cell. (**, P<0.01)*


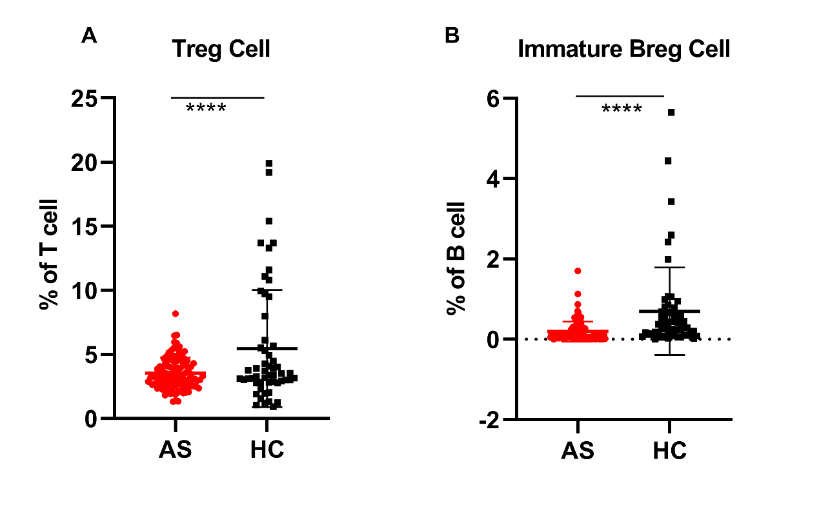


***Supplementary Fig 2.*** ***The proportion of regulatory lymphocytes is shown for both the AS and HC groups.*** *P-value summary: (*, P<0.05) (**, P<0.01) (***, P<0.001) (****, P<0.0001). Treg cell: Regulatory T cell, Breg cell: Regulatory B cell.*

***Supplementary Table 5. Demographic and disease characteristics of AS patients treated with Anbainuo.***

|  | Baseline | After Anbainuo Therapy | P |
| --- | --- | --- | --- |
| Demographics |  |  |  |
| Sex (M: F) | 19:4 | - | - |
| Age (years) | 30 (25-36) | - | - |
| D D (years) | 8.00 (4.50-13.00) | - | - |
| Disease status |  |  |  |
| CRP (mg/L) | 12.10 (2.60-20.90) | 2.50 (0.50-8.00) | 0.015 |
| ASDAS-CRP | 2.97±1.02 | 1.37±1.04 | **** |
| BASDAI | 4.25±1.37 | 1.69±1.32 | **** |

*Results are displayed as mean ± standard deviation for normal distribution data and median (IQR) for non-normally distributed data. Anbainuo: Recombinant Human Tumor Necrosis Factor-α Receptor Ⅱ: IgG Fc Fusion Protein for Injection, made in China. M: male, F: female. DD: disease duration. CRP: C-reactive protein. BASDAI: Bath Ankylosing Disease Activity Index. ASDAS: Ankylosing Spondylitis Disease Activity Score. (**, P<0.01)*
